# Molecular Docking: Shifting Paradigms in Drug Discovery

**DOI:** 10.3390/ijms20184331

**Published:** 2019-09-04

**Authors:** Luca Pinzi, Giulio Rastelli

**Affiliations:** Department of Life Sciences, University of Modena and Reggio Emilia, Via Giuseppe Campi 103, 41125 Modena, Italy

**Keywords:** molecular docking, drug discovery, drug repurposing, reverse screening, target fishing, polypharmacology, adverse drug reactions

## Abstract

Molecular docking is an established in silico structure-based method widely used in drug discovery. Docking enables the identification of novel compounds of therapeutic interest, predicting ligand-target interactions at a molecular level, or delineating structure-activity relationships (SAR), without knowing *a priori* the chemical structure of other target modulators. Although it was originally developed to help understanding the mechanisms of molecular recognition between small and large molecules, uses and applications of docking in drug discovery have heavily changed over the last years. In this review, we describe how molecular docking was firstly applied to assist in drug discovery tasks. Then, we illustrate newer and emergent uses and applications of docking, including prediction of adverse effects, polypharmacology, drug repurposing, and target fishing and profiling, discussing also future applications and further potential of this technique when combined with emergent techniques, such as artificial intelligence.

## 1. Introduction

The experimental screening of large libraries of compounds against panels of molecular targets, i.e., High-Throughput Screening (HTS), has represented the gold standard for discovering biologically active hits. However, the high costs required to establish and maintain these screening platforms often hamper their use for drug discovery [1]. Moreover, considering the recent developments in computer technology and the rapid increase of structural, chemical, and biological data available on an ever-growing number of therapeutic targets, it is easily understandable how the use of in silico approaches as chemoinformatics, molecular modeling, and artificial intelligence (AI) has significantly increased in the last decades [2,3,4,5,6]. Indeed, in silico approaches now enable the virtual screening of millions of compounds in an affordable time, thus reducing the initial costs of hit identification and improving chances of finding the desired drug candidates. At present, several molecular modeling techniques are available to facilitate drug discovery tasks, most of them being classified into structure-based and ligand-based approaches.

Structure-based methods rely on the information derived from the knowledge of the 3D structure of a target of interest, and they allow ranking databases of molecules according to the structural and electronic complementarity of ligands to a given target [7]. In this context, molecular docking is among one of the most popular and successful structure-based in silico methods, which help predict the interactions occurring between molecules and biological targets [7]. This process is generally accomplished by first predicting the molecular orientation of a ligand within a receptor, and then estimating their complementarity through the use of a scoring function [7].

Since its first appearance in the mid-1970s, docking has proved to be an important tool to help understanding how chemical compounds interact with their molecular targets, and for drug discovery and development. As a matter of fact, the number of studies reporting: (i) the use of molecular docking to identify structural determinants necessary for efficient ligand-receptor binding, and (ii) the development of more accurate docking methods, have heavily increased since its first appearance [7,8,9,10,11,12,13,14,15,16,17,18,19,20,21]. Among the first and more interesting studies on the use of docking in drug discovery and biology is the one from Kuntz et al. in the early 1980s [13]. In this study, the authors described a computational method enabling the exploration of geometrically feasible ligand-receptor alignments for the known heme-myoglobin/metmyoglobin and thyroxine/prealbumin structures [13]. This study was not the first to employ docking for predicting potential conformations of molecular complexes [9]. However, it reported for the first time the use of a simplified function containing solely the terms “hard sphere repulsions” and “hydrogen bonding” to describe protein-ligand interactions, which strongly differed from previous studies [9,11,12,22]. Moreover, the authors were also the first to consider the receptor as a solid rigid body, whose binding site is constituted by “pockets”. Interestingly, the method adopted in this study was able to predict structures close to those of already reported X-ray complexes, and also to find protein conformations that could be used for energy refinement and eventually design novel ligands [13]. Since then, molecular docking underwent dramatic improvements, for example, by employing flexible algorithms in the calculations [21,23,24,25,26]. Moreover, it also started to be used for the design and optimization of compounds with therapeutic interest. An example of this comes from a study of Ring et al., in which several structure-based drug design methods, including docking, were performed to identify novel non-peptidic inhibitors of enzymes of the serine and cysteine protease families [27]. The results achieved in this work further consolidated the use of computer-aided structure-based drug-design methods for assisting the development of lead compounds [27].

Given the potential offered by this method, increasing efforts have been directed towards the improvement of docking algorithms and for overcoming its intrinsic limitations [28,29,30]. Indeed, major limitations characterizing docking include a restricted sampling of both ligand and receptor conformations in pose prediction, and the use of approximated scoring functions, which very often provide results that do not correlate with the experimental binding affinities [31,32]. Nevertheless, the application of docking in drug design is limited to biological targets for which crystal structures are known. Several approaches have been adopted to overcome this latter limitation. For example, the unavailability of 3D structures is often bypassed by building homology models derived from structural templates with highly-homologous sequences. Moreover, these methods could also be used in tandem with molecular dynamics (MD) to further validate and refine the in silico modeled complexes [33,34,35]. Nevertheless, the recent progress in structural biology and crystal structure determination, which are progressively increasing the accessibility to experimentally derived ligand-target complexes [36,37,38,39], will certainly mitigate this issue. In silico strategies, including molecular dynamics, have also been widely used to explore the conformational space of the investigated targets, ligands, and ligand-target complexes, and thus better describing the dynamic behavior of ligand-target complexes and refining the docking results [34,40,41]. More rigorous virtual screening methodologies have also been developed to improve the docking-based ligand-target complex predictions [42,43,44,45,46]. Indeed, these post-docking refinement and rescoring methods are of great interest in drug discovery because they usually provide higher hit rates in virtual screening campaigns and allow better correlation with experimental data [44,46].

**Figure 1:**
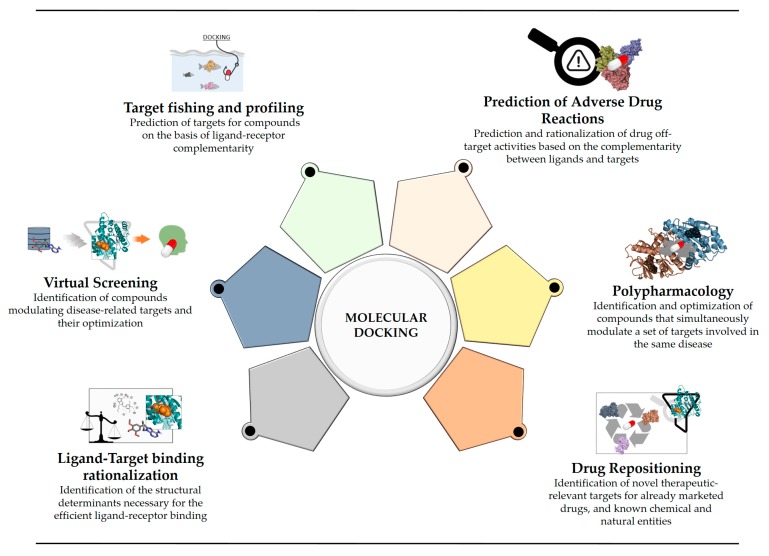
Main applications of molecular docking in current drug discovery. Molecular docking is currently employed to help rationalizing ligands activity towards a target of interest and to perform structure-based virtual screening campaigns, similarly to as when it was first developed. Besides these applications, it can also be used to identify series of targets for which the ligands present good complementarity (target fishing and profiling), some of them being potentially responsible for unexpected drug adverse reactions (off-targets prediction). Moreover, docking is also currently employed for the identification of ligands that simultaneously bind to a pool of selected targets of interest (polypharmacology) and for identifying novel uses for chemical compounds with already optimized safety profiles (drug repositioning).

A number of reviews discussing the role and applications of docking, and the possibilities it could offer in drug design and development, have been reported [7,18,47,48,49,50]. However, it should be noted that the uses and applications of docking have been changing since its first appearance. In fact, although it was first developed to investigate molecular recognition between large and small molecules, it is now also widely used to assist different tasks of drug discovery programs, such as hit identification and optimization, drug repositioning, *a posteriori* target identification (reverse screening), multi-target ligand design, and repositioning (Figure 1) [49,51,52,53,54,55,56,57,58]. Moreover, docking allows understanding the relationships between different molecular targets involved in a given disease, which is also of high relevance for polypharmacology [59] and modern drug discovery in general.

In particular, the use of this technique has broadened towards novel drug discovery horizons, fueled by the improvement of docking algorithms and by the increase of the publicly accessible information on ligands and targets. For example, thanks to the improved speed and prediction power, docking has also been embedded into large-scale screening protocols to identify [60], e.g.: (i) protein binding sites in which ligands could bind [61]; (ii) novel molecular targets of known ligands [54]; (iii) potential adverse drug reactions (ADRs) [62], and; (iv) ligands with novel chemotypes active against a given target or a set of desired targets [55].

In this review, we will discuss how docking methods have been used to help assisting drug discovery tasks, giving particular emphasis on recent drug design strategies, including polypharmacology, drug repurposing, target identification, and prediction of adverse drug reactions.

## 2. Current Rational Design Approaches, Including Docking 

**Figure 2:**
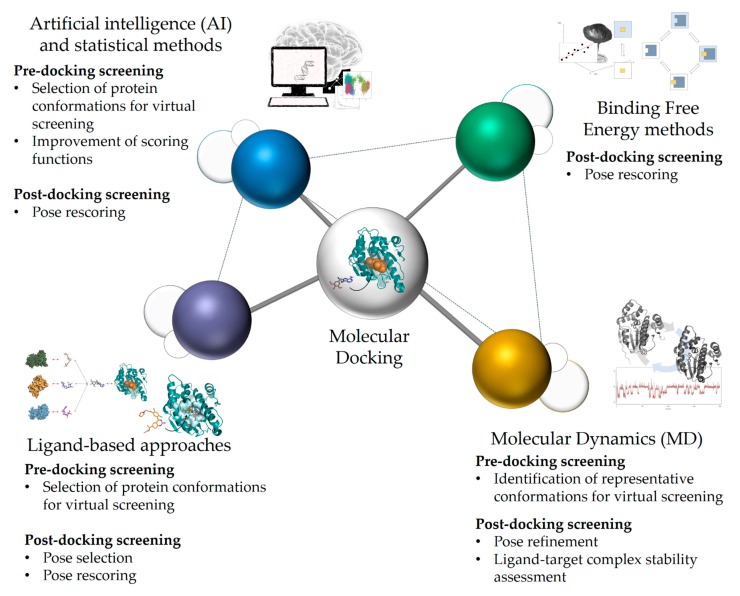
Integration of docking with ligand-based, molecular dynamics, binding free energy approaches, artificial intelligence (AI), and statistical methods. According to the available information, different in silico approaches can be combined with docking to generate integrated workflows with improved prediction performances. Different approaches can also be combined to integrate docking (e.g., molecular dynamics and binding free energy estimations can be combined with docking to improve virtual screening results). Likewise, different approaches can also be applied at different phases of the screening workflow to improve docking predictions. For example, molecular dynamics could be combined with AI-based methods to identify suitable receptor conformations for docking. Then, ligand-based approaches could be applied for rescoring the predicted docking poses [50,65,66].

The possibilities offered by molecular docking in drug discovery are well established [3,5,7,18,47,49,50,63]. However, docking presents intrinsic limitations that limit its prediction performances, the most relevant being reported in the previous section. Although docking has been mainly used as a standalone method for drug design, it is now often integrated into workflows that include other computational methods, such as ligand-based, structure-based, and AI approaches (Figure 2) [50,64]. This helps to account for some of the most relevant limitations characterizing this structure-based method.

In particular, ligand-based approaches have been used to select suitable protein conformations for docking screenings [67,68,69,70]. The ability of docking to discriminate active compounds from decoys can strongly depend on the used protein structures and the similarity degree of the screened ligands with those co-crystallized in the employed target conformations [69,70,71,72]. In this regard, Broccatelli et al. recently reported a study in which different ligand-based methods have been applied for the selection of protein conformations for docking, comparing the performance of different protocols in retrieving known CDK2 inhibitors within two distinct datasets [69]. Similar considerations arose also from the more recent studies of Xu et al. [73] and Kumar et al. [74], in which the authors successfully predicted the affinity and binding mode of a series of Hsp90 [73] and farnesoid X receptor ligands [74], by combining ligand-based approaches with docking.

Ligand-based approaches have also been used to improve the prediction performance of docking screenings, e.g., by measuring the 3D similarity between the binding conformation predicted by docking and the experimental conformation of the ligand co-crystallized in the employed protein conformation [75]. For example, Perryman et al. [76] demonstrated that pharmacophore-based rescoring can improve docking predictions in blind virtual screenings. Similar results were also obtained by Jiang et al. [77], who retrospectively evaluated the performance of DOCK [78,79] against three clinical drug targets (EGFR, IGF-1R, and HIVgp41). According to the obtained in silico results, in particular, the authors demonstrated that the combination of the standard DOCK energy function with an especially devised pharmacophore-based scoring function outperformed single approaches in discriminating active from inactive compounds [77]. Similarly, 3D- and shape-based similarity have also been used in tandem with docking for pose selection and virtual screening [80,81]. In particular, Kumar et al. demonstrated that 3D shape similarity matching is a promising approach for the selection of docking poses [81]. Moreover, they also observed that docking-based scoring could be conveniently combined with 3D shape similarity to improve virtual screening results [81]. Anighoro et al. recently explored an alternative path to force field-based scoring functions to improve the ranking of candidate ligands of four therapeutic drug targets (DHFR, GR, VEGFR2, and HIV1PR) [80]. According to the obtained in silico results, in particular, the authors demonstrated that the use of 3D similarity approaches for rescoring docking poses could improve ranking lists and hit-rates in structure-based virtual screenings [80]. Nevertheless, methods based on the comparison of ligand-target binding features, e.g., through the use of the protein-ligand interaction fingerprints (PLIFs), have also successfully been explored for rescoring docking results [82,83,84].

Standing on current literature data, the combination of ligand-based and structure-based approaches allows to heavily improve the prediction power, and thus hit-rates, in virtual screening campaigns. However, it should also be noted that the possibility to apply ligand-centered methods in tandem with docking could be explored solely for those targets that have at least one reported co-crystallized ligand [70,81].

Structure-based approaches, such as molecular dynamics and binding free energy estimations, have also widely been used in combination with docking to improve virtual screening results. In particular, MD allows to evaluate residues flexibility in the target binding site, as well as to explore larger conformational changes potentially accessible to a given protein [33,34,41]. Therefore, it represents an efficient tool to identify receptor conformations for docking [70,85] and to evaluate the stability of the predicted complexes [33,34]. The possibilities offered by MD in prospective in silico screening are particularly appealing for flexible targets with a limited number of reported crystallographic conformations. An example of this comes from a study of Wang et al. [86], who have performed classical MD simulations in explicit solvent to evaluate the stability of the α-helical structure of amyloid β42 (Aβ42), thus identifying a representative protein conformation to perform virtual screening of commercially available compounds [86]. This approach allowed the selection of a set of compounds to be experimentally validated, five of them showing inhibition of Aβ42 aggregation in the micromolar range. Moreover, one of the identified hits also displayed inhibition of BACE1, which plays a key role in the pathogenesis of Alzheimer’s disease [86]. Similar considerations could also be drawn from a more recent work of Spyrakis et al. [87], who demonstrated that standard molecular dynamics, clustering, and linear discriminant analysis (LDA) can be integrated to substantially improve structure-based virtual screening results. In this study, in particular, the authors firstly performed MD simulations on three flexible targets (the purine nucleoside phosphorylase PNP, the A_2A_ receptor, and the ABL1 tyrosine-protein kinase) in search of novel protein conformations. Then, they performed clustering via the K-medoids method on the calculated molecular dynamics trajectories [88] to identify MD-derived representative conformations of the investigated targets. Finally, the performance of the FLAP docking program [89] in discriminating active from inactive compounds extracted from DUD-e [90], which is a database useful to benchmark and validate docking protocols, was assessed. LDA was used to automatically select the best combination of protein templates among MD-derived representative and experimentally observed structures yielding the best screening results. 

Altogether, the discussed examples demonstrated how the inclusion of classical MD in docking-based protocols could improve virtual screening performances, especially when dealing with highly flexible targets [86,87]. More advanced enhanced sampling techniques, such as umbrella sampling [91], metadynamics [92], and replica exchange MD [93], can also be applied to identify protein conformations for docking screening. Indeed, these techniques, which allow exploring a protein conformational landscape far larger with respect to that of standard MD simulations, have already been applied to study protein flexibility and function [94,95,96,97,98,99,100,101] and to identify additional binding pockets that could be exploited for the design of novel inhibitors [102,103]. However, it should be noted that the application of these advanced methods is computationally more demanding with respect to standard MD. 

Combinations of docking with standard molecular dynamics and binding free energy estimations have also been recently explored to account for protein flexibility and to improve virtual screening predictions, respectively [44,46,104,105,106]. In fact, results of currently available docking algorithms might be affected by poor conformational sampling [7,31]. Moreover, they might provide inaccurate binding energy estimations [7,23] derived by approximate scoring functions [28,107,108]. Indeed, several scoring functions based on different algorithms and concepts, which can be classified into empirical, knowledge-based, and force field-based, have been developed for docking so far [28,30]. However, all of them employ a series of mathematical functions with approximations that do not accurately take into account some thermodynamic elements of the binding energy (e.g., entropy changes upon binding and solvation effects), to allow the fast prediction of ligand-target complex affinity [28,30]. Several approaches have been adopted to account for these issues so far. For example, Rastelli et al. [42] developed BEAR (Binding Estimation After Refinement), a post-docking tool that first performs MD-based structural refinement of ligand-protein complexes, and then predicts their binding free energy with the MM-PBSA and MM-GBSA methods [109]. Indeed, several studies, showing the improvements that the MM-PBSA and MM-GBSA methods, and BEAR provided, both in *a posteriori* and prospective virtual screenings, have been reported [44,46], demonstrating that the application of these approaches heavily improves docking results.

The more advanced and computationally expensive free energy prediction methods Free Energy Perturbation (FEP), Thermodynamic Integration (TI), and funnel metadynamics can also be used for the post-processing of docking results [110,111,112]. For example, Lee et al. [113] successfully applied a computational workflow that integrates molecular docking, MD simulation, and FEP calculations to predict the binding mode and ligand-protein affinity of a series of MDM2 and MDMX inhibitors, discussing also virtues and vices of the adopted protocol. More recently, Bhati et al. [114] proposed a method that combines MD and TI and was able to provide accurate binding affinities. The performance of their protocol was validated on five well-characterized proteins involved in several physiological processes. However, although the latter approaches are more accurate than docking scoring functions, or even other free energy methods as MM-PBSA and MM-GBSA, in predicting ligand-protein affinity, they are computationally expensive, therefore potentially less suitable for the screening of large libraries of compounds [115].

Very recently, statistical and Artificial Intelligence approaches have also gained a foothold in drug discovery [64]. In fact, these methods allow to easily exploit the ever-growing source of information contained in publicly available structural, chemical, and bioactivity databases, leading to more accurate binding affinity predictions. In particular, machine learning (ML) approaches, including Random Forest (RF) [116] and Support Vector Machines (SVM) [117], have been applied for improving the docking-based binding affinity predictions [29,118]. For example, Xie et al. [119] successfully developed and applied a computational workflow integrating SVM-based classification with docking calculations to identify novel inhibitors of the c-Met tyrosine kinase. In particular, the authors firstly developed and validated an SVM classification model able to discriminate between active and inactive c-Met ligands. Then, they compared the performances of the developed SVM-based model with respect to those of docking, and then the combination of the two methods. The combined SVM-model/docking approach, which provided the best hit-rates and enrichment factors, was finally used to screen a large library of ligands, leading to the identification of eight active compounds among those selected [119]. SVM techniques were also implemented in ensemble schemes to improve docking predictions on flexible targets. For example, Leong et al. [120] recently reported the development and retrospective validation of tailored SVM classification models able to improve docking posing and scoring predictions against the N-methyl-D-aspartate GluN1 receptor. In particular, the authors firstly developed SVM-based posing models by performing docking calculations on ligands reported in complex with seven crystal structures of the N-methyl-D-aspartate GluN1 receptor. Then, they derived a series of SVM-based scoring models from the poses predicted with the same procedure, for a dataset of compounds with reported bioactivity data. Finally, the authors performed a retrospective validation of the newly developed SVM models by screening a further set of compounds, demonstrating that their approach outperformed standard docking in accurately predicting ligand poses and binding affinities [120].

Machine learning approaches have also been used to improve docking scoring functions. For example, Ballester et al. [121] developed among one of the first ML-based scoring function, called *“RF-Score*”, which uses Random Forest to improve protein-ligand binding affinity predictions. In particular, the authors firstly developed an RF-based scoring function by using different sets of ligand-protein complexes with known activity data reported within the PDBbind database [122]. Then, they compared the performance of “*RF-Score*” with that of other sixteen scoring functions implemented in currently available docking programs, demonstrating that it improved docking results both in virtual screening and lead optimization tasks and that its performances are independent from the employed training sets [121]. Similar considerations come also from a more recent study of Wang et al. [123], who developed a novel scoring function by re-parameterizing the one already implemented in AutoDock Vina [124], with random forest. According to the achieved results, the authors demonstrated that their method outperformed standard docking programs in predicting scores that correlate with experimentally derived binding affinities. Moreover, they also demonstrated that the prediction performances of their scoring function improved with the use of larger datasets for the RF-based scoring function correction [123]. 

Deep learning (DL) approaches have also been studied to improve docking results. For example, Pereira et al. [125] recently reported an approach based on Convolutional Neural Networks (CNN) called “*DeepVS*”, which learns the features relevant for the binding of a ligand to a target under study, given a set of docking results. In particular, they firstly developed a scoring function that generates DL-based docking scores based on structural data describing a ligand-protein complex (i.e., the atom types and their partial charges, distance between the atoms, and the amino acid types). Then, to demonstrate that the developed scoring function can provide improved results, the authors performed extensive validation screenings on forty different datasets reported in the DUD-e database [90]. According to the reported results, the newly developed “*DeepVS*” scoring function was able to provide higher screening performances with respect to those of standard scoring functions. Interestingly, this study was the first to report the application of deep learning for rescoring docking poses without requiring human-defined parameters, thus making it a particularly appealing approach for unsupervised, large scale, virtual screening campaigns [125].

Less sophisticated, but still robust, statistical techniques, such as score distribution data analysis, have also been examined. In particular, Wang et al. [126] recently reported the application of score distribution data analysis and docking for protein structure selection. Moreover, the adopted approach, which can be employed for a variety of virtual screening purposes, also allowed to successfully identify off-targets activities for 43 anticancer drugs approved by Food and Drug Administration (FDA) [126].

Although the recent introduction of statistical techniques allowed improving results of docking screenings, their applicability heavily depends on the availability of structural, chemical, or bioactivity data. Therefore, such approaches may not represent the optimal choice to improve docking prediction performances when dealing with recently identified, or not yet thoroughly studied, therapeutic targets. 

## 3. Reverse Screening for Target Fishing and Profiling

Docking has also been recently used for a variety of other purposes in drug discovery. In particular, Reverse Docking (RD), which allows predicting the biological targets of a molecule of interest [127], represents a valuable approach for computational target fishing and profiling [60].

Several docking approaches and algorithms are available to enable the reverse screening of a ligand towards a library of protein structures and to assess their binding affinity. However, the application of these approaches requires suitable libraries of targets [60,127]. Indeed, several databases are currently available to help performing RD screenings. Among one of the most known databases to facilitate computational target identification is PDTD [128], which provides information about protein structures, diseases, biological functions, and drugs. Moreover, tailored libraries of targets can also be manually built upon publicly available databases of crystal structures and binding pockets, such as the Protein Data Bank (PDB) [129], sc-PDB [130], Pocketome [131], and Therapeutic Target Database (TTD) [132]. In particular, the PDB [129] and TTD [132] databases represent well-known reservoirs of information developed to help facilitating computational, molecular and structural biology, and to provide data about targets and diseases, respectively. The sc-PDB [130] and Pocketome [131] databases were instead developed for comparing protein cavities, better describing the ligand-protein pharmacophoric properties and for target identification via pocket-based virtual screening, and to benchmark docking screenings, respectively. Although these libraries of targets were not specifically developed for target fishing and profiling, they allow covering large structural spaces of the known proteome. However, it should be noted that the preparation of such libraries is a time-consuming task because each structure in the databases requires to be properly prepared for the docking calculations [60,127]. 

In RD screenings, potential targets of a ligand can be ranked according to scoring functions implemented in commonly used docking programs as Glide [133,134]. For example, Park and Cho [135] performed an extensive RD screening on a library of disease-related proteins, including entries from PDTD and around 500 kinases, to identify potential targets for 26 ginsenosides. Results of the screenings allowed the authors to identify potential ligand-protein interactions for some of the investigated natural products with anticancer targets, such as MEK1, EGFR, and Aurora A [135]. Interestingly, although the predicted ligand-protein interactions were not experimentally validated, anticancer activity has been reported for some of the investigated ginsenosides [136]. Moreover, the authors identified potential interactions with targets as acetylcholinesterase, human Carbonic Anhydrase II, and glutamate dehydrogenase, which might be responsible for cholinergic side effects and nephrotoxicity [135]. Although being extensively used in RD screenings, results of standard docking scoring functions can heavily depend on the investigated targets, or even on the employed structural conformations of a given protein. Therefore, normalization of the scores is generally recommended to avoid target-dependent biased results [60,127,137]. 

Integration of docking with more sophisticated ML-based methods has also very recently been explored for target predictions [138,139]. An example is a study of Nogueira and Koch [139], in which SVM and Neural Networks (NN) models were used to improve the results of reverse docking screenings, pre-processed with the PADIF (Protein Atom Score Contributions Derived Interaction Fingerprint) method [82]. In particular, the authors firstly built datasets of compounds for twenty biological targets with already reported bioactivity data on ChEMBL [140] and X-ray crystal structures. Then, for each target, they developed SVM and NN machine learning models able to discriminate active from non-active compounds based on the docking-derived PADIFs. Finally, they retrospectively validated their models, achieving notable prediction performances, both in terms of target ranking and on multi-target selectivity predictions [139]. Although these structure-based approaches might present several advantages over ligand-based methods and standard docking, they are time and computationally demanding. Moreover, they also need a large amount of bioactivity data to train the models, which sometimes could not be available for some of the targets under study. However, considering the recent advances in hardware and software [141,142] and the increased amount of publicly available bioactivity data, ML-based approaches will certainly play a central role in future target identification and profiling tasks. 

Consensus approaches based on RD have also recently been reported for target fishing [143]. In particular, Lapillo and coworkers [143] performed an extensive benchmarking study on 13 different RD screening procedures to identify which method performed better in predicting targets for known ligands. Moreover, they also explored a docking-based consensus approach to improve RD target prediction performances [143]. According to the results reported in this study, target-fishing performances of the different docking procedures ranged around 25% and 35% (in terms of true predictions) for single approaches, and 36% for the consensus. However, this study also showed that the results of the applied RD approaches could be dependent on the features of the investigated proteins binding site [143].

As the set-up of reverse docking screening workflows requires more efforts and longer preparation with respect to standard virtual screening, various tools and web platforms have also been recently developed to facilitate RD. Most of them entrust in already compiled libraries of disease-relevant targets and implement standard programs (e.g., DOCK [79], AutoDock [144], and AutoDock Vina [124]) for performing reverse docking calculations. Therefore, they enable researches to easily identify the biological targets of their molecules of interest, even without massive computational efforts. Among the most relevant programs and web platforms currently in use for docking-based reverse screening are INVDOCK [145], TarFisDock [54], ACTP [146], and idTarget [147], which have been widely employed for target fishing and profiling in several studies, and for different purposes [148,149,150,151,152]. In particular, INVDOCK [145] is a software based on docking, which has been devised for the identification of potential targets for drugs, candidates under clinical trials and ligands, and to facilitate the study of their potential side effects. TarFisDock [54], ACTP [146], and idTarget [147] are web platforms that enable remote RD screening of a given ligand to a set of protein structures. In particular, TarFisDock [54] and idTarget [147] enable the screening of a given ligand to a set of proteins available within the PDTD and PDB by using the DOCK [79] and MEDock [153] docking programs, respectively. Similarly, the ACTP [123] web server enables the screening of a ligand by using the Libdock docking protocol [154]. However, reverse docking analyses of this latter web platform are restricted to a set of autophagy-related protein targets [146].

The utility of these platforms for drug discovery has already been reviewed elsewhere [60,127]. However, considering the relevance of the topic and the improvements in:

(i) currently available computational techniques and software in general, which allow to more accurately screen larger databases;
(ii) hardware facilities, which enable a faster screening of ligands to targets, to a larger public, and;
(iii) crystallography [36,37,38,39] and homology modeling [155,156] techniques, which allow expanding our knowledge on structural biology;

we envision that further advances in reverse docking will certainly play a central role for target fishing and profiling of ligands in future drug discovery.

## 4. Prediction of Adverse Drug Reactions

The early identification of drug side effects is of high interest in drug discovery. In fact, it is well known that most of the drug candidates fail clinical trials because of side effects deriving from unexpected interactions with off-targets. Moreover, post-marketing side effect analyses on approved drugs (i.e., pharmacovigilance) are also important because they allow revealing potential safety risks that often could not be detected within clinical trials [157]. Several computational approaches are currently available to assist this task [158,159,160,161]. However, most of them require a satisfactory amount of bioactivity data, or of already reported adverse effects as an input for the model training [158,159,160,161]. Interestingly, molecular docking needs solely the structural information of the targets to perform its predictions. Therefore, it represents a valuable approach to predict potential side effects of compounds at early phases of clinical and pre-clinical developments, or on marketed drugs with not yet reported exhaustive drug labels and bioactivity records. Indeed, applications of RD screening for identifying drug adverse effects have already been reported in the literature [127,149,162,163,164]. For example, Ji et al. [149] performed RD screenings to identify potential side effects for a series of anti-HIV drugs, highlighting several proteins whose modulation has already been associated with adverse reactions. Indeed, more than 85% of their target predictions found correspondence with clinical evidence reported in the literature [149]. However, it should also be noted that some of the targets known to be modulated by the investigated anti-HIV drugs could not be predicted within their study, due to the lack of structural information in the analyzed database [149] or because of intrinsic limitations of the adopted screening approach. Altogether, the results reported in this study demonstrated that docking-based reverse screening can be an efficient tool for identifying putative adverse drug reactions. However, the obtainment of good performance predictions is tightly related to the information reported in the analyzed database of targets. Databases developed to facilitate adverse reactions recognition for drugs have already been reported, SIDER being one of the best known [165]. In particular, SIDER contains more than 140,000 drug-ADR pairs registered in clinical trials and post-marketing surveillance [165], which could facilitate the identification of target activities responsible for side effects. However, databases of targets can also be developed on purpose for performing more focused RD-based ADR analyses, as previously discussed for target fishing and profiling.

More sophisticated screening approaches based on consensus docking [166], or combination with machine learning techniques [138], have also been explored to improve the predictions of drug adverse events. For example, Jaundoo et al. [166] recently applied a consensus docking approach using three different programs, namely AutoDock, AutoDock Vina, and Glide, to predict putative adverse reactions of a set of 43 drugs approved by FDA for the treatment of Gulf War Illness (GWI)-related symptoms. Indeed, the authors performed their analyses to identify side effects potentially arising by the combined use of some of these drugs, suggesting caution for some of them. Moreover, strengths and limitations related to the use of consensus approaches with respect to standard docking were also discussed by the authors [166].

Combinations of docking with statistical approaches have been applied for predicting off-target activities of already reported drugs (see above) [126]. Moreover, a combination of docking with machine learning models has recently been explored. For example, Luo et al. [138] performed an extensive study on more than 1200 compounds extracted from DrugBank against 600 human proteins for predicting or rationalizing drug adverse effects. In particular, the authors firstly performed docking calculations with AutoDock Vina for assessing binding modes and affinity scores of the ligand-protein complexes, and they built ML-based models upon adverse reactions data reported within the SIDER database [165]. Then, they performed side effect predictions by comparing the evaluated ligand-protein scores with the trained ML models. Their analyses resulted in the prediction of 1533 putative adverse reactions and provided potential explanations on the biological mechanisms behind some of already reported drug side effects (e.g., irinotecan that induces decreased libido) [138], demonstrating that ADRs predictions on a large scale are feasible with computational workflows that integrate docking.

Based on the results of the aforementioned studies, it can be argued that consensus approaches with other molecular modeling methods, or integration with AI approaches, will play a pivotal role in future docking-based side effects prediction, allowing to overcome the main limitations currently affecting docking programs.

## 5. Polypharmacology

To avoid potentially harmful side effects, the pharmaceutical industry focused on the development of highly selective drugs. However, the high attrition rates in the late stages of clinical trials due to a lack of therapeutic efficacy have moved modern drug design towards polypharmacology, which refers to the identification of ligands that hit a set of selected, therapeutic-relevant targets [57,59,167,168]. In this context, molecular docking can provide valuable opportunities because it allows the identification of chemical scaffolds that efficiently and simultaneously bind to a pool of selected targets of interest. Indeed, several studies related to the use of docking for the design of novel multi-target ligands have already been reported [53,169,170]. Moreover, its utility for *de novo* polypharmacology design has also been reviewed [57,168,171,172]. The design of multi-target ligands on rational grounds is challenging [57]. Moreover, the selection of protein conformations to be used for docking can heavily affect the success of the design [70]. This is especially true when dealing with targets with structurally distant binding sites. Considering how difficult it can be to design multi-target ligands, docking is now generally applied in combination with other in silico approaches. In particular, several studies reporting the identification of multi-target ligands are based on the combination of docking screening with pharmacophore modeling [53,169,172]. For example, we recently reported the identification of the first Hsp90/B-Raf dual inhibitors, demonstrating that sub-structure pre-filtering and pharmacophore-guided docking can be efficiently combined to search for polypharmacology ligands that bind to structurally unrelated targets [53]. However, workflows integrating docking with other in silico techniques have also been pursued for *de novo* multi-target drug design and polypharmacology in general [70,171,173]. For example, Selvam et al. [173] recently reported a study in which a combination of MD, probe mapping, and docking approaches was applied to investigate the selectivity of multi-target ligands towards a set of bioaminergic G-protein-coupled receptors [173]. The results showed that combined workflows, including docking, can be used to guide the design of selective, potentially safer, multi-target molecules able to circumvent side effects commonly associated with antipsychotic drugs. Moreover, the study also demonstrated how different in silico approaches could be efficiently combined to identify structural peculiarities of disease-related targets, for which selective ligands are not yet reported [173].

Web tools and platforms based on docking are also available to explore polypharmacology and for identifying multi-target activities of ligands, such as the Computational Analysis of Novel Drug Opportunities (CANDO) platform [174,175], DRAR-CPI [176], and DPDR-CPI [177]. In particular, CANDO is a multicomponent platform, including molecular docking, that allows predicting potential multi-target interactions of compounds, whereas DRAR-CPI [176] and its upgraded version DPDR-CPI [177] are two web servers that allow identifying candidate targets for a given molecule, and also enable drug repositioning. 

Considering the number of computational tools available for the in silico screenings and the challenges that must be faced in a multi-target drug discovery campaign, the best combination of methods should be selected, case by case, upon the available data on the targets [57,172], and hardware and software facilities.

## 6. Drug Repositioning

Drug repositioning, or repurposing, represents an established drug discovery approach that allows identifying novel therapeutic uses for already approved drugs, candidate compounds under clinical evaluation, natural products, or already synthesized ligands in general [178]. Given the wealth of information reported on ligands, targets and diseases into publicly available databases, increasing efforts have been made on the application of in silico repositioning-based discovery strategies over the last decades. Indeed, in silico repositioning approaches have already demonstrated to provide novel valuable opportunities for drug discovery and development [65,179,180,181,182,183]. 

In this context, molecular docking has become among one of the most popular computational approaches to repurpose compounds towards novel therapeutic targets. For example, docking can be applied in reverse screening approaches to identify novel molecular targets for known ligands, based on their structural complementarity [127,184]. Docking allows virtually screening databases of approved drugs, natural products, or already synthesized compounds into one or more biological targets of interest in an affordable time. An example of this comes from a study of Kinnings et al., who performed extensive structure-based studies on nine different *Mycobacterium tuberculosis* InhA structures to evaluate whether the entacapone and tolcapone drugs, approved for the treatment of Parkinson’s disease, might be repurposed against tuberculosis [51]. Their results allowed the identification of entacapone as a promising lead compound against resistant strains of *Mycobacterium tuberculosis* [51]. Moreover, this study also demonstrated that the same drug could be potentially used for the treatment of unrelated disorders, e.g., Parkinson’s disease and tuberculosis [51,185,186]. On the same line, Dakshanamurthy et al. [187] recently performed extensive docking-based virtual screenings on a subset of compounds taken from the DrugBank [188,189], BindingDB [190], and FDA (https://www.fda.gov/) databases against several X-ray crystal structures of human proteins reported in the Protein Data Bank [129]. According to the reported results, the authors discovered that the anti-parasitic drug mebendazole is also an anti-angiogenic VEGFR2 inhibitor. Moreover, they also successfully discovered that the COX-2 inhibitor celecoxib and dimethyl celecoxib bind to Cadherin-11, which is a protein mediating calcium-dependent cell-cell adhesion that plays a crucial role in rheumatoid arthritis [187].

Despite their great potential for drug discovery [191,192,193], naturally occurring molecules have very seldom been explored for drug repositioning. Indeed, repositioning strategies based on the integration of chemocentric target identification with docking analyses have been recently explored for identifying novel therapeutic uses of natural compounds. For example, we have recently searched for novel therapeutic targets for the two non-psychoactive cannabinoids cannabigerol (CBG) and cannabichromene (CBC), by integrating shape-based similarity screening with rigid and flexible docking calculations [53]. In particular, in this study, a computational shape-based similarity screening was firstly performed within the DrugBank database. This analysis allowed the identification of InhA, which is an enzyme studied for the development of anti-tubercular drugs, as a potential target for repositioning of both CBG and CBC. Then, extensive docking analyses of the two cannabinoids were performed in the InhA binding site. Interestingly, docking calculations predicted that CBG, but not CBC, is a good candidate for the inhibition of InhA, a finding that was later confirmed by subsequent experimental *in vitro* assays. Altogether, results of this approach, which can be applied to repurpose both natural products and synthetic ligands, clearly demonstrate how molecular docking and ligand-based methods could complement each other in providing more accurate drug repositioning predictions [53]. The combined application of docking with machine learning approaches has also been explored for drug repurposing [194]. Moreover, molecular docking has also been efficiently integrated into web-based tools to enable remote structure-based virtual screenings of given libraries of compounds [195,196,197] and for drug repurposing predictions [176,177]. For example, Lagarde et al. [196] recently reported a retrospective repurposing screening on a curated library of already approved drugs by using the MTiOpenScreen docking web service [195]. In particular, the authors firstly built three different libraries of purchasable compounds containing approved drugs (“*Drugs-lib*”), food constituents (“*FOOD-lib*”), and natural products (“*NP-lib*”). Then, they performed docking screenings with the MTiOpenScreen web service into the developed “*Drugs-lib*” library to evaluate whether the adopted protocol was able to identify five approved ligands, for which drug reposition against cancer-related targets have already been reported. The performed analyses were able to identify the investigated drugs within the first 1500 top-scored ligands in all of the performed screening campaigns, demonstrating that the adopted protocol might be efficiently used to repurpose already marketed compounds [196].

Based on these promising results, docking represents a valuable approach also for predicting new therapeutic indications for already approved drugs, natural compounds, and already synthesized ligands, especially when used in tandem with other computational methods, such as ligand-based similarity approaches.

## 7. Concluding Remarks

Since its first appearance in the mid 1970′s, molecular docking has represented a unique in silico tool to assist drug design and discovery. However, beyond the applications for which it was originally developed, docking is now also widely employed to assist a variety of other drug discovery tasks, such as the identification of novel chemical scaffolds within large libraries of compounds, to perform in silico target fishing and profiling for drug repositioning, polypharmacology, prediction of adverse effects and beyond, as described in this review article. Being a versatile tool, docking will certainly find application also in other fields of drug discovery. Moreover, docking has been successfully embedded within automated workflows for the screening of large libraries of compounds and targets [54,145,146,147,195,198]. Of course, the recent advancements in the field of high-performance computing played a key role in this respect. For example, they enabled the in silico screening of millions of compounds in an affordable time [141,142]. Moreover, the recent advancements on Graphics Processing Units (GPUs) have also provided remarkable improvements, both in data-driven drug discovery and in molecular dynamics simulations [34,199]. Indeed, GPU calculations enabled a large exploration of the conformational landscape potentially accessible to proteins, in shorter times with respect to CPUs [200]. Finally, GPU computing made big data-driven computation tasks accessible to a larger public [201], and it is expected to play a prominent role, not only in docking but in future in silico drug design in general [202]. 

The modalities by which docking is used to assist the different tasks of drug discovery have also changed along the years. In particular, although it was initially developed and used as a standalone method, docking is now mostly employed in combination with other computational approaches within integrated workflows. This allows to overcome some of the most relevant intrinsic limitations characterizing molecular docking, such as the non-exhaustive conformational sampling and the use of approximate scoring functions [7,31,32,46]. The application of combined approaches usually results in improved prediction performances and allows to better exploit the information coming from different sources. Indeed, applications of combined workflows, including docking, have been explored to assist different tasks of drug discovery. For example, docking has been used in tandem with ligand-based, molecular dynamics, binding free energy calculations, and AI approaches to improve the prediction performances in *de novo* virtual screening, as well as to assist target fishing, ADRs prediction, polypharmacology, and drug repurposing, as discussed. However, one should be aware of the fact that each computational method has its limitations, which might hamper its integration with docking in combined workflows, or even reduce the prediction power of the adopted protocol. For example, methods that provide predictions upon already available bioactivity and chemical data (e.g., AI techniques) may not be the best approach to integrate with docking for less-characterized molecular targets. Likewise, ligand-based approaches might not represent the optimal choice for improving docking predictions when a sufficient number of ligands for the target(s) under study is not available. However, when the combination is feasible, these methods demonstrated to heavily improve docking predictions, both in terms of hit-rates and enrichment factors. On the contrary, molecular dynamics and binding free energy estimations could help improving docking predictions even for less-characterized targets, for example, through the identification of conformational ensembles to be used for structure-based analyses [85], and to more accurately evaluate the ligand-protein binding affinity [46]. Considering the number of in silico tools and techniques currently available, there are still countless opportunities for docking to be explored in integrated workflows. Moreover, their integration will also be facilitated by the continuous improvements in hardware and software engineering, as discussed. Besides, novel valuable opportunities for data and methods integration will certainly come along the increase of the publicly available structural, chemical, and biological information, and its implementation within databases, web platforms, and automated workflows. Further efforts should be directed toward a better integration of the different approaches with the publicly available information reported in these databases. This is expected to provide novel valuable opportunities in future drug discovery and development and, in particular, in the design of challenging and innovative drugs (i.e., multi-target ligands), as well as in assisting ligand profiling and repositioning. Considering the high attrition rates characterizing drug discovery [203,204], the possibilities offered by docking in combination with the approaches outlined here will be important to reduce time and costs in both the development of clinical candidates with better safety profiles (target profiling and ADRs findings) and for the identification of novel applications of already known drugs (target profiling and drug repositioning).

## Abbreviations

ADR	Adverse Drug Reaction
AI	Artificial Intelligence
BEAR	Binding Estimation After Refinement
CANDO	Computational Analysis of Novel Drug Opportunities
CBC	Cannabichromene
CBG	Cannabigerol
CNN	Convolutional Neural Networks
CPU	Central Processing Unit
DL	Deep Learning
FDA	Food and Drug Administration
FEP	Free Energy Perturbation
GPU	Graphics Processing Units
GWI	Gulf War Illness
HTS	High-Throughput Screening
LDA	Linear Discriminant Analysis
MD	Molecular Dynamics
ML	Machine Learning
NN	Neural Networks
PADIF	Protein Atom Score Contributions Derived Interaction Fingerprint
PLIFs	Protein-Ligand Interaction Fingerprints
PNP	Purine Nucleoside Phosphorylase
RD	Reverse Docking
RF	Random Forest
SAR	Structure-Activity Relationships
SVM	Support Vector Machines
TI	Thermodynamic Integration
